# Supplementary material for: Proximity-magnetized quantum spin Hall insulator: monolayer 1 T’ WTe2/Cr2Ge2Te6
Source: Nat Commun. 2022 Sep 1;13:5134. doi: 10.1038/s41467-022-32808-w (PMC9436961; doi:10.1038/s41467-022-32808-w)
Supplement: Supplementary file 1 — Supplementary Information [file 41467_2022_32808_MOESM1_ESM.pdf]

Supplementary Information for

Proximity-magnetized quantum spin Hall insulator: monolayer 1T' $WTe_2/Cr_2Ge_2Te_6$

Junxue Li[1,2]†, Mina Rashetnia[1]†, Mark Lohmann[1], Jahyun Koo[3], Youming Xu[4], Xiao Zhang[5], Kenji Watanabe[6], Takashi Taniguchi[7], Shuang Jia[5], Xi Chen[4], Binghai Yan[3], Yongtao Cui[1], and Jing Shi[1]*

1. *Department of Physics and Astronomy, University of California, Riverside, CA 92521, USA*
2. *Department of Physics, Southern University of Science and Technology, Shenzhen 518055, China*
3. *Department of Condensed Matter Physics, Weizmann Institute of Science, Rehovot, Israel*
4. *Department of Electrical and Computer Engineering, University of California, Riverside, CA 92521, USA*
5. *International Center for Quantum Materials, School of Physics, Peking University, Beijing 100871, China*
6. *Research Center for Functional Materials, National Institute for Materials Science, 1-1 Namiki, Tsukuba 305-0044, Japan*
7. *International Center for Materials Nanoarchitectonics, National Institute for Materials Science, 1-1 Namiki, Tsukuba 305-0044, Japan*

†These authors contributed equally to this work

*Corresponding author Email: jing.shi@ucr.edu

Contents

1. Optical characterization of monolayer (ML) WTe_2 and optical images of ML-WTe_2/CGT devices after fabrication

2. Induced anomalous Nernst effect (ANE) vs. spin Seebeck effect (SSE)

3. Heating-power dependence of ANE signal

4. Experimentally measured and COMSOL simulated temperature difference in ANE device

5. H_z-dependent ANE signals from channels 4-7 and 4-5 at selected temperatures

6. Anisotropic magnetoresistance (AMR) vs. spin Hall magnetoresistance (SMR)

7. Activation energy extracted from the Bulk-only channel of ML-WTe_2

8. Theoretical calculations of band structure, Berry curvature, AHE conductivity and ANE coefficient of ML-WTe_2 on CGT

9. Gate voltage dependence of 1f resistance and 2f voltage in device D1 at 4 K

10. Separation of bulk and edge ANE signals

11. Effect of low-temperature thermal conductivity on ANE magnitude

1. Optical characterization of monolayer (ML) WTe_2 and optical images of ML-WTe_2/CGT devices after fabrication

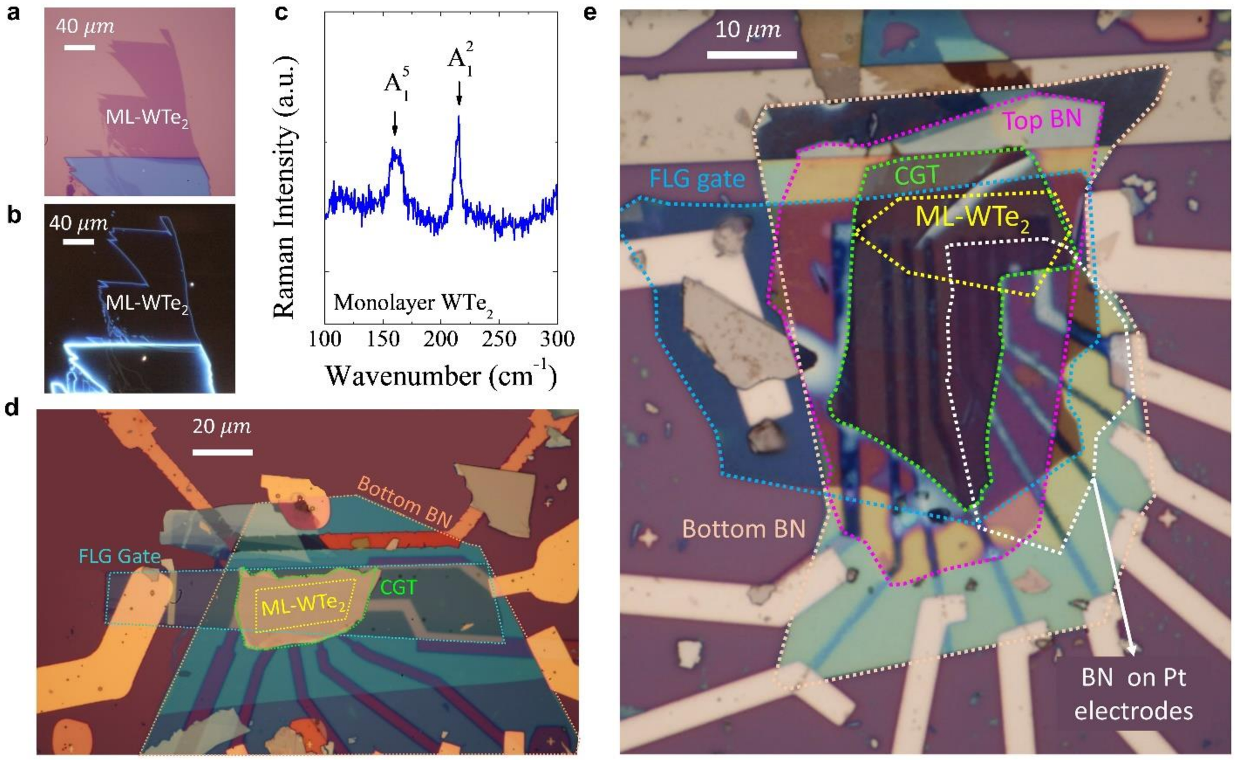

Fig. S1. Optical characterization of monolayer (ML) WTe_2 and optical images of ML-WTe_2/CGT devices after fabrication. **a**, Bright-field image of exfoliated ML-WTe_2; **b**, Dark-field image of exfoliated ML-WTe_2. **c**, Raman spectrum of bare ML WTe_2 exfoliated on SiO_2 substrate. The laser wavelength is 532 nm. **d**, Optical image of device D1 after transfer of ML-WTe_2/CGT heterostructure on to the Pt electrodes. A thin BN layer around 35 nm is encapsulated on the top of the device later for protection. **e**, Optical image of the device D7 after fabrication. All the layers are identified by the dashed polygons.

We exfoliate ML WTe_2 flakes in a glove box and identify them based on the optical contrast and color. Figs. S1a and S1b show the bright-field and dark-field optical images, respectively. It is clear that the freshly exfoliated ML WTe_2 flake is very clean with no visible particles on top. We characterize ML WTe_2 flakes using Raman (as shown in Fig. S1c). Two peaks located at 162.1 cm^{-1} and 214.2 cm^{-1} are identified as Raman mode A_1^5 and A_1^2, respectively, which is consistent with the reported characteristic Raman spectrum of ML WTe_2 (1). Here we also show the

optical images of ML-WTe_2/CGT devices D1 and D7 after the fabrication is complete (as presented in Figs. S1d and S1e). The fabrication details are summarized in the Methods section.

2. Induced anomalous Nernst effect (ANE) vs. spin Seebeck effect (SSE)

In well-studied ferromagnetic insulator (FMI)/heavy metal (HM) heterostructures, there has been a vigorous debate about the origin of the transverse voltage response to a temperature gradient. Two possible mechanisms can give rise to such a magneto-thermoelectric voltage hysteresis: Anomalous Nernst effect (ANE) due to induced ferromagnetism in HM as a result of the proximity effect and spin Seebeck effect (SSE) which does not require any induced ferromagnetism in HM. It is usually very difficult to exclude either one. In general, SSE voltage is generated by spin-charge conversion in the HM layer with strong spin-orbit coupling (SOC) (1) capable of producing the inverse spin Hall effect (ISHE) signal (3, 4). The thermally generated spin current in the FMI layer flows into the HM layer driven by the vertical component of the temperature gradient ∇T. This out-of-plane spin current is converted to a lateral charge current $\boldsymbol{J_c} \sim \nabla T \times \boldsymbol{\sigma}$ and then an ISHE voltage which depends on the spin polarization direction $\boldsymbol{\sigma}$ determined by the magnetization of the FMI. For a vertical temperature gradient component, the ISHE voltage should only be sensitive to the in-plane magnetization of the FMI or the in-plane component of $\boldsymbol{\sigma}$. Here in ML-WTe_2/CGT, the SSE mechanism can be straightforwardly excluded as briefly explained below. If the in-plane magnetization component determines the ISHE signal due to a vertical ∇T, the SSE voltage would vanish at high out-of-plane magnetic fields H_z when the magnetization is saturated along the z-direction. In the hysteresis loops shown in Fig. 1d, the signal saturates at two different nonzero values at large H_z. There is no apparent in-plane magnetization characteristic present. Instead, the signal closely resembles the out-of-plane magnetization M_z loop. In our devices, the nonlocal heater produces a dominant in-plane ∇T component. Even if a sizable out-of-plane temperature gradient exists, it does not generate an observable SSE signal.

For ANE, the competing mechanism in the presence of ∇T, the thermoelectric field is proportional to $\nabla T \times \boldsymbol{M}$. Under an in-plane temperature gradient, the ANE signal is only sensitive to the out-of-plane magnetization component M_z. Clearly, the ANE mechanism matches the observed voltage characteristics. However, the ANE only exists in conductive materials. In WTe_2/CGT heterostructures, the CGT layer is at least 4 orders of magnitude more resistive than

WTe_2, and the low-temperature transfer process does not make conductive. CGT does not generate any thermoelectric signal. Hence, the ANE signal must originate from the magnetized WTe_2 layer. The M_z-like characteristics of the observed loops in Fig. 1d are consistent with the ANE mechanism in the presence of an in-plane temperature gradient generated by the nonlocal heater.

3. Heating-power dependence of ANE signal

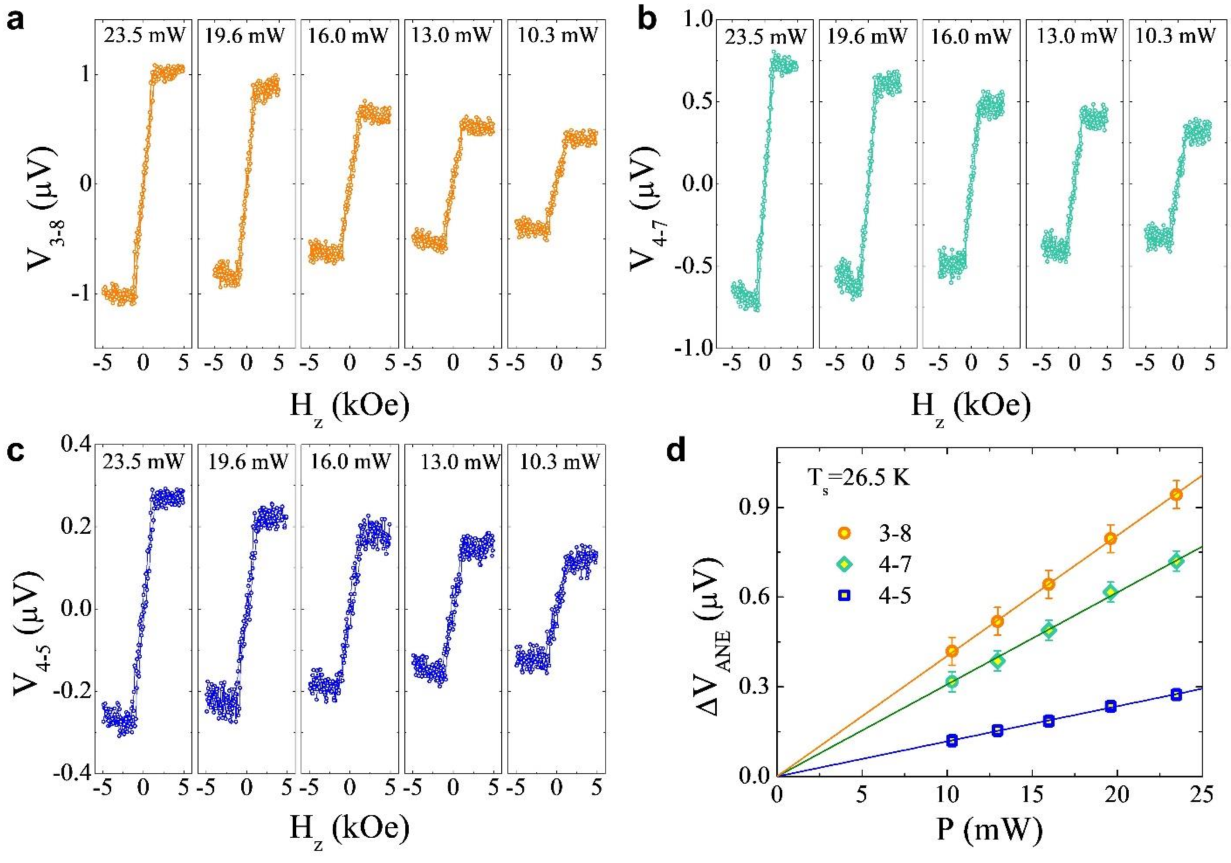

Fig. S2. Heating power dependence of anomalous Nernst effect (ANE) signal from ML-WTe_2/CGT heterostructure. a, **b**, **c**, ANE voltages from channel 3-8 (**a**), 4-7 (**b**) and 4-5 (**c**) as a function of out-of-plane magnetic field under different heating power. The sample temperature is fixed at 26.5 K by keeping resistance of ML-WTe_2 a constant during the measurements. **d**, Heating power dependence of ANE voltages from 3-8, 4-7 and 4-5 channels, the sample temperature is fixed at 26.5 K. The straight lines are linear fitting results.

To further confirm the thermoelectric nature of ANE signal in Fig. 1d, we perform heating-power dependence of the ANE signals from channels 3-8, 4-7 and 4-5 of device D1. Under fixed system temperature, the sample temperature rises due to Joule heat from nonlocal heater. To obtain the heating-power dependence of ANE signal at a fixed sample temperature, we adjust the system temperature accordingly to maintain the targeted sample temperature (T_s=26.5 K) monitored by the sample resistance. As summarized in Fig. S2, the hysteresis loops from channel 3-8(Fig. S2a), 4-7(Fig. S2b) and 4-5(Fig. S2c) shrink as the heating-power decreases. Clearly, the linear power dependence displayed in Fig. S2d entails a linear temperature gradient ∇T-dependence, and thus is consistent with the ANE responses.

4. Experimentally measured and COMSOL simulated temperature difference in ANE device

∇T is an important parameter that determines the value of transverse Seebeck coefficient $S_{yx}(=\frac{\left[\frac{\Delta V_y}{l_y}\right]}{\nabla_x T})$, where ΔV_y is the transverse voltage, i.e., the ANE voltage, generated by a temperature gradient $\nabla_x T$ along the x- or longitudinal-direction, l_y is the channel length in the transverse direction. Here we demonstrate that $\nabla_x T$ can be determined using both experimental method and finite element simulations.

Experimentally we use the resistance of heater and ML-WTe_2 to calibrate the actual temperatures at their respective locations. The resistances as a function of temperature are measured with a very low current density first, which serves as a temperature calibration curve. As the heater is turned on, local temperatures rise above the system temperature, which can be accurately monitored by the resistance. We use this resistive thermometry to determine the actual temperatures of the heater and ML-WTe_2. The heater temperature, as well as the temperature of ML-WTe_2, increase as the heating power P increases (as shown in the right panel of Fig. S3a), the actual temperature of the heater (and ML-WTe_2) is determined using the R vs. T calibration curve (left-panel of Fig. S3a). In our measurements of P-dependent ANE voltages (as shown in Fig. S2), the actual sample (ML-WTe_2) temperature is held at T_s =26.5 K which is monitored by its resistance while adjusting the measurement system temperature. At the constant T_s, the

temperature gradient at the sample location is different for different heating power levels. This effect can be seen in the temperature difference ΔT between the heater T_{heater} and T_s for different P's and their linear relation in Fig. S3b. Fig. S3c shows that the ΔT-normalized ANE voltages measured in three channels are nearly constant, which is consistent with Fig. S3b. For $l_y(4-7) \approx 15\ \mu m$, assuming a linear temperature decay between the heater and sample, we use $\nabla_x T = \frac{\Delta T}{15\ \mu m}$ and obtain $S_{yx}(4-7) \approx 0.037\ \frac{\mu V * \mu m}{K}$.

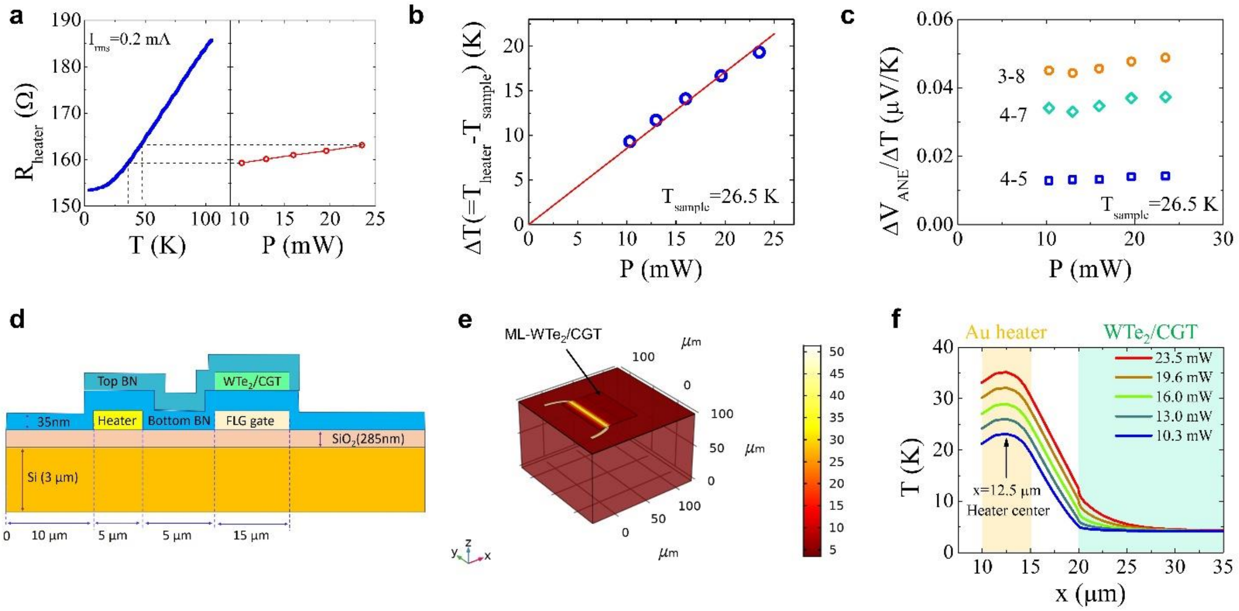

Fig. S3. Experimental and simulated temperature difference in the anomalous Nernst effect (ANE) device. a, Heater resistance as a function of temperature and heating power in device D1. AC current with rms magnitude from 8 mA to 12 mA is applied in the nonlocal heater to produce different heating-power. **b**, Heating-power dependence of temperature difference ΔT between nonlocal heater and ML-WTe_2, where the sample temperature is fixed at 26.5 K during the measurements. **c**, ΔT-normalized ANE magnitudes from three different channels as a function of nonlocal heating power. **d**, Cross section of ANE device structure used in the COMSOL simulation for heat conduction. **e**, Simulated 3D distribution of the temperature in the ANE device. **f**, Simulated temperature under different heating powers as a function of lateral distance x. The position of Au heater and ML-WTe_2/CGT device is marked with yellow and green shades.

In actual devices, the local temperature near the heater is not a linear function of the distance; therefore, we use a finite element method (COMSOL) to simulate the temperature profile in our ANE device. Based on the device configuration (Fig. S3d), the width and thickness of the Au heater are 5 μm and 50 nm, respectively. The heater sits on top of a 285 nm thick SiO_2 on a Si substrate. The WTe_2/CGT heterostructure composite layer is separated from the FLG by a BN flake. The overall dimensions of the entire device are 160 μm ×160 μm ×100 μm. A 3D view of the simulated domain is shown in Fig. S3e. A constant temperature boundary condition of 4 K is applied to the bottom and four side surfaces of the Si substrate. We use experimentally measured resistivity of Au film in the simulation of heating generated by heater. Due to the large electrical resistance of SiO_2 and BN in contact with the Au wire, the electric current in the heater is converted to a uniformly distributed heat flux along the Au wire. The temperature-dependent heat capacities, thermal conductivities and interfacial thermal conductivities of relevant materials are obtained from literatures (5-21). Fig. S3f shows the temperature profile of the ANE device for different heating-power. It is clear that the temperature profile is highly nonlinear. The local temperature gradient at 27.5 μm (the center of WTe_2/CGT heterostructure) extracted from the simulation is -0.1742 $\frac{K}{\mu m}$ for P=23.5 mW, which is ~11.6 times smaller than the global average temperature gradient (30.3 K/15 μm) assuming a linear temperature distribution. Therefore, we greatly underestimate the value of S_{yx} by assuming a linear temperature distribution, and the actual S_{yx} should be one order of magnitude greater than the value determined from the experimentally measured local temperatures assuming a linear temperature profile.

5. H_z-dependent ANE signals from channels 4-7 and 4-5 at various temperatures

To investigate correlation between the observed ANE signals and ferromagnetic order in CGT layer, we carry out detailed temperature-dependent measurements. Figure 1e summarizes the ANE signal from channel 3-8 at various sample-temperatures ranging from 14.9 K to 67.4 K. Here, we plot the ANE signals from channels 4-7 (Fig. S4a) and 4-5 (Fig. S4b) as a function of H_z at the same selected temperatures. We can clearly see that the characteristics from both channels are similar: the size of the hysteresis loop shrinks as the sample warms up, and the hysteresis loop vanishes above the Curie temperature of the CGT crystal (T_c=61 K), which is

also summarized in Fig. 1f. These features strongly suggest that the ANE voltage of ML-WTe_2 stems from the proximity-induced ferromagnetism by the adjacent ferromagnetic insulator CGT.

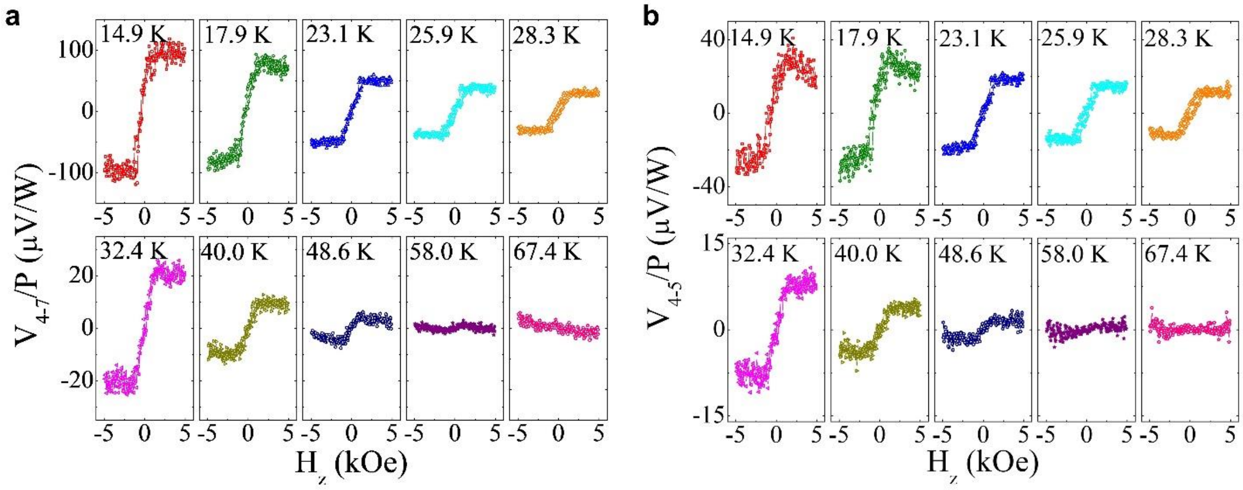

Fig. S4. a, **b**, **H_z-dependent ANE signals from 4-7 (a) and 4-5 (b) channels at various sample temperatures ranging from 14.9 K to 67.4 K.** All the ANE voltages are normalized to heating-power, sample temperature is calibrated using resistance versus temperature curve.

6. Anisotropic magnetoresistance (AMR) vs. spin Hall magnetoresistance (SMR)

In FMI/HM heterostructures such as YIG/Pt, there has been a long-standing debate on the origin of observed magnetoresistance and AHE. In ferromagnetic metals, the resistance depends on the relative orientation between the current and magnetization. This is widely known as the anisotropic magnetoresistance or AMR. In FMI/HM, it is possible to have AMR when the HM interface layer is magnetized. This effect quickly decays as the HM thickness increases, which makes it difficult to ascertain this is the only or dominant mechanism. The same magnetized interface layer in HM can give rise to the AHE. On the other hand, the spin Hall magnetoresistance (SMR) effect was proposed to be wholly or partly responsible for the observed magnetoresistance in FMI/HM because the spin current generation in and reflection across the HM layer thickness produce the magnetization-dependent resistance involving both spin Hall and inverse spin Hall effects (22). The same mechanism was used to explain observed AHE in such heterostructures. An importance difference is that the spin current mechanism does

not require induced ferromagnetism in HM while the AMR effect must originate from a ferromagnetic source.

Our ML-WTe_2/CGT heterostructures can serve as a test system to differentiate the two aforementioned mechanisms. Since the spin current across the one atomic layer cannot bend the trajectories of spin-up and -down carriers to generate the spin Hall and inverse spin Hall effects, the SMR mechanism cannot operate here despite the strong SOC in WTe_2. On the other hand, there is only one atomic layer in WTe_2, the induced magnetization in the interface layer does not decay and the AMR or AHE signals do not get diluted by the presence of other unmagnetized layers. Additionally, the SMR mechanism can be refuted from the H_z-dependence of the $1f$ MR signal with the following arguments. As H_z is swept below the saturation field H_s, CGT starts to evolve to multi domains as previously imaged by magnetic force microscopy (23) and thus develop in-plane components, M_x and/or M_y. According to the SMR theory (22), the finite M_x component does not produce any resistance change, whereas M_y only causes the resistance to decrease from its saturation value as M is aligned with the z(-z) direction. This is obviously contradictory to the peak feature in our $1f$ MR signal. Hence, the $1f$ MR signal is clearly the AMR effect expected for the magnetized ML-WTe_2. In addition, the $1f$ AHE signal, i.e., the field-antisymmetric component, provides another proof of the induced ferromagnetism in ML-WTe_2. Similar proximity-induced AHE has also been observed in other quantum materials including graphene (24) and topological insulators (25, 26).

7. Activation energy extracted from the Bulk-only channel of ML-WTe_2

As shown in Fig. S5, the conductance of the Bulk-only channel (5-6) is much smaller than that of the Bulk+ Edge channel (13-14), indicating that the edge channel starts to dominate the transport properties at low temperatures. The conductance of ML-WTe_2 increases as the device is warmed up, indicating an energy gap and activated transport properties. We extract the activation energy $\boldsymbol{\Delta}$, or energy gap, through the Arrhenius fit of the form $G \propto e^{-\frac{\Delta}{k_B T}}$, where $k_B (= 0.086\ meV/K)$ is the Boltzmann's constant. Fig. S5 displays the Arrhenius fitting results of device D7. We obtain $\Delta \approx 3.16\ meV$ by fitting the data from Bulk-only channel from 2 K to

40 K, over which the edge conduction dominates. The activation gap we extracted is very close to the value reported in recent literature (27).

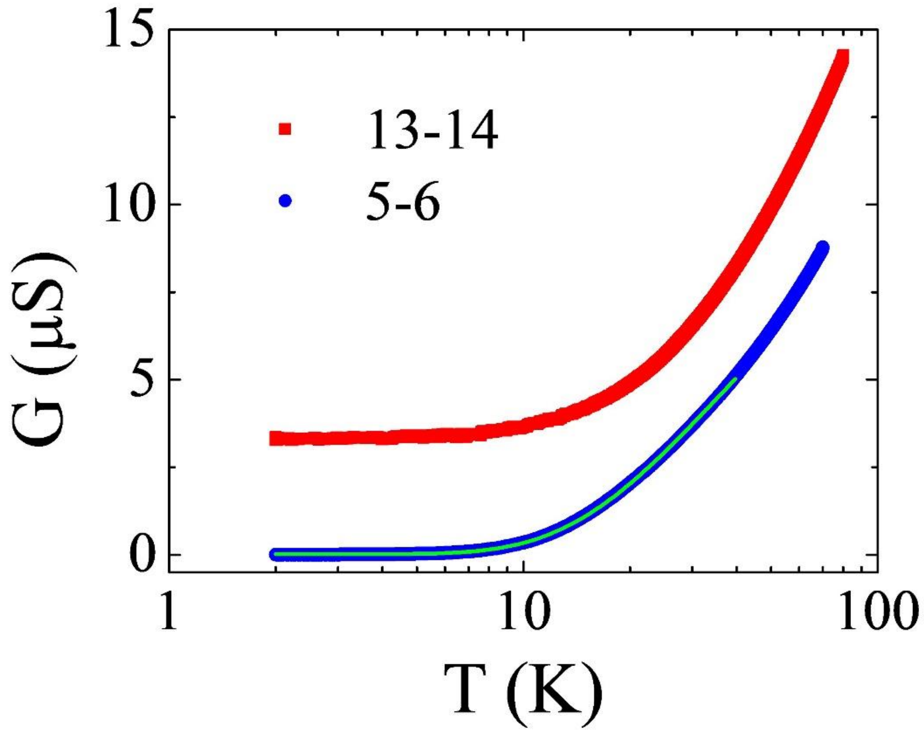

Fig. S5. Extraction of activation energy Δ from device D7. The green curve is Arrhenius fitting result. The fitting range is from 2 K to 40 K, the activation gap is 3.16 meV.

8. Theoretical calculations of band structure, Berry curvature, AHE conductivity and ANE coefficient of ML-WTe_2 on CGT

We have simulated ML-WTe_2 on CGT by ab initio density-functional theory (DFT) calculations. The ML-WTe_2 was reported to be a quantum spin Hall insulator (QSHI). CGT is a ferromagnetic insulator, where the Cr atom has a magnetic moment of $3\mu_B$. Because of the proximity to the ferromagnetic CGT layer, the ML-WTe_2 exhibits ferromagnetism with a small spin splitting in the band structure. Consequently, ML-WTe_2 acquires finite Berry curvature (see

Fig. S7). We employ GGA+U (U=3 eV) calculations so that ML-WTe_2 shows a gap in the band structure.

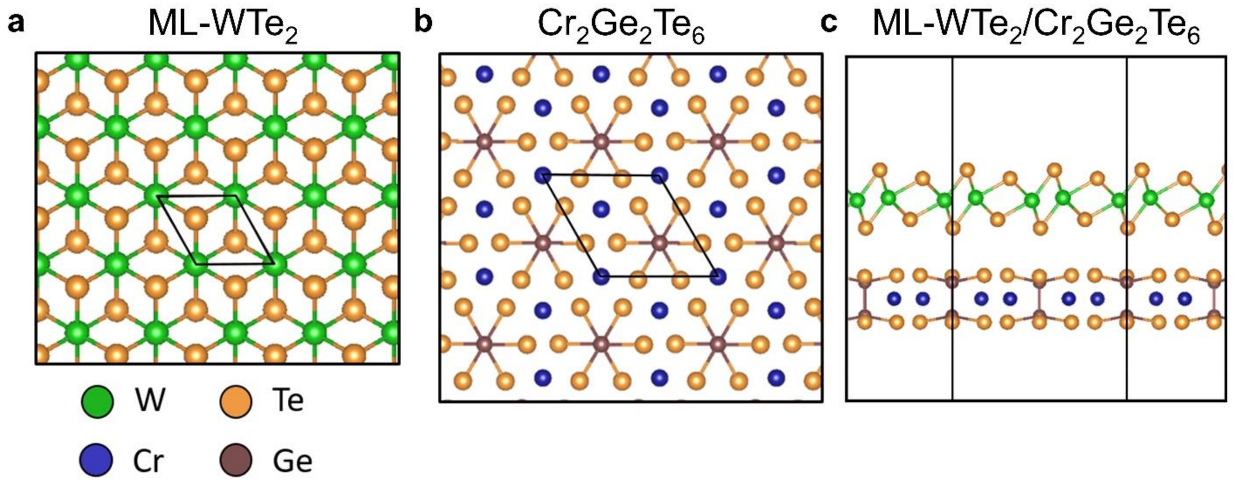

Fig. S6. **Atomic structure of ML-WTe2, $Cr_2Ge_2Te_6$, and their heterostructure. a**, Top view of the atomic structure of ML-WTe_2. **b**, Top view of the atomic structure of $Cr_2Ge_2Te_6$ (CGT). **c**, Side view of stacked atomic structure of WTe_2 on CGT substrate. The black rhombuses in **a** and **b** indicate the unit cell of each structure.

We adopt a WTe_2/CGT bilayer structure to simulate the magnetic proximity effect. A rectangular shaped supercell includes 2×1 unit-cell WTe_2 (see Fig. S6). The atomic structure optimization is fully performed. Based on the stacked bilayer structure, we perform DFT calculations following the framework of the generalized gradient approximation (28) with Hubbard U (GGA+U) with the Vienna *ab-initio* package (29). We employ the PBE-D2 method to describe the vdW interaction (30). Spin-orbit coupling (SOC) is included in all calculations. To perform the anomalous Hall conductivity calculation, we have projected the DFT results based on the Bloch wave functions into the Wannier functions to construct an effective Hamiltonian ($\hat{H}$) to evaluate the Berry curvature (Ω^z_{xy}), the anomalous Hall conductivity(σ^z_{xy}), and anomalous Nernst conductivity(α^z_{xy}).

$$\sigma^z_{xy}(\mu) = -\frac{e^2}{\hbar}\int_{Bz}\frac{dk}{(2\pi)^3}\sum\nolimits_{\varepsilon_n<\mu}\Omega_{xy} \qquad \text{(S1)}$$

$$\Omega_{xy}^{z}(k) = -i\sum_{m\neq n}\frac{\langle n|\hat{v}_x|m\rangle\langle n|\hat{v}_y|m\rangle-(y\leftrightarrow x)}{(\varepsilon_n(k)-\varepsilon_m(k))^2} \qquad \text{(S2)}$$

$$\alpha_{xy}^{z}(T,\mu) = -\frac{1}{e}\int d\varepsilon\frac{\partial f(\varepsilon-\mu,T)}{\partial\varepsilon}\frac{\varepsilon-\mu}{T}\sigma_{xy}^{z}(\varepsilon). \qquad \text{(S3)}$$

Here μ is the chemical potential, ε_n is the eigenvalue of the $|n\rangle$ eigenstate, and $\hat{v}_i = \frac{d\hat{H}}{\hbar dk_i}$ is the velocity operator. A k-point of grid of 200×200×1 is used for the numerical integration.

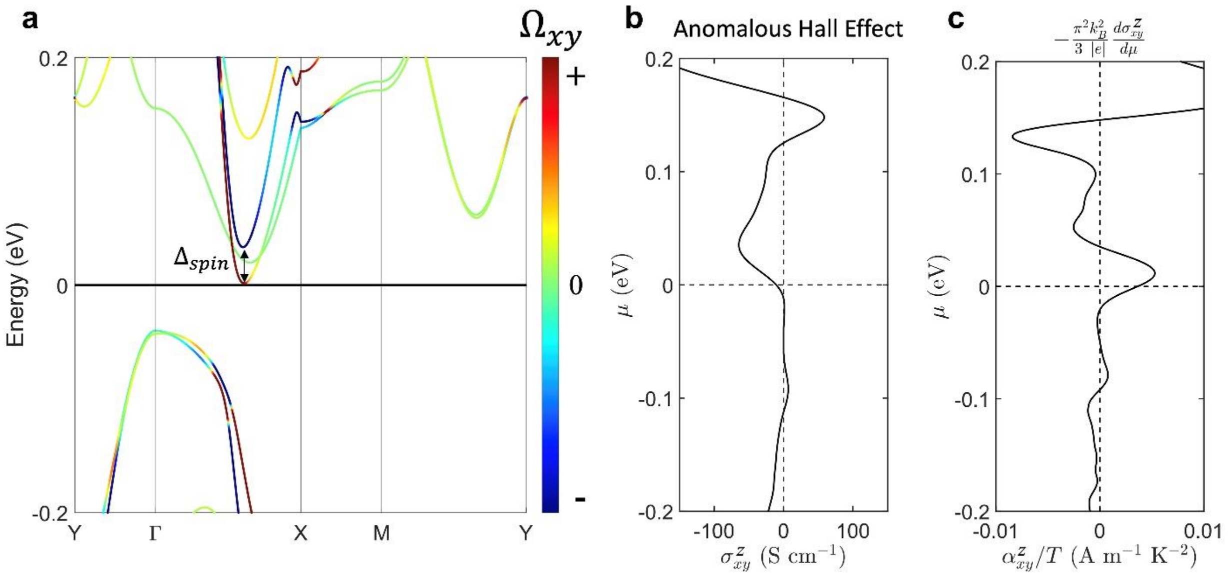

Fig. S7. Band structure, anomalous Hall conductivity and anomalous Nernst coefficient of ML-WTe_2 on $Cr_2Ge_2Te_6$. a, Calculated band structure with the Berry curvature (Ω_{xy}) of ML-WTe_2 stacked on CGT by GGA+U (U=3 eV). The Fermi energy is located at zero. The spin splitting (Δ_{spin}) due to CGT proximity in the conduction band is more than 30 meV. **b**, Calculated anomalous Hall conductivity (σ_{xy}^{z}) as a function of the chemical potential (μ). **c**, Calculated anomalous Nernst coefficient (α_{xy}^{z}/T) as a function of μ at the low temperature limit.

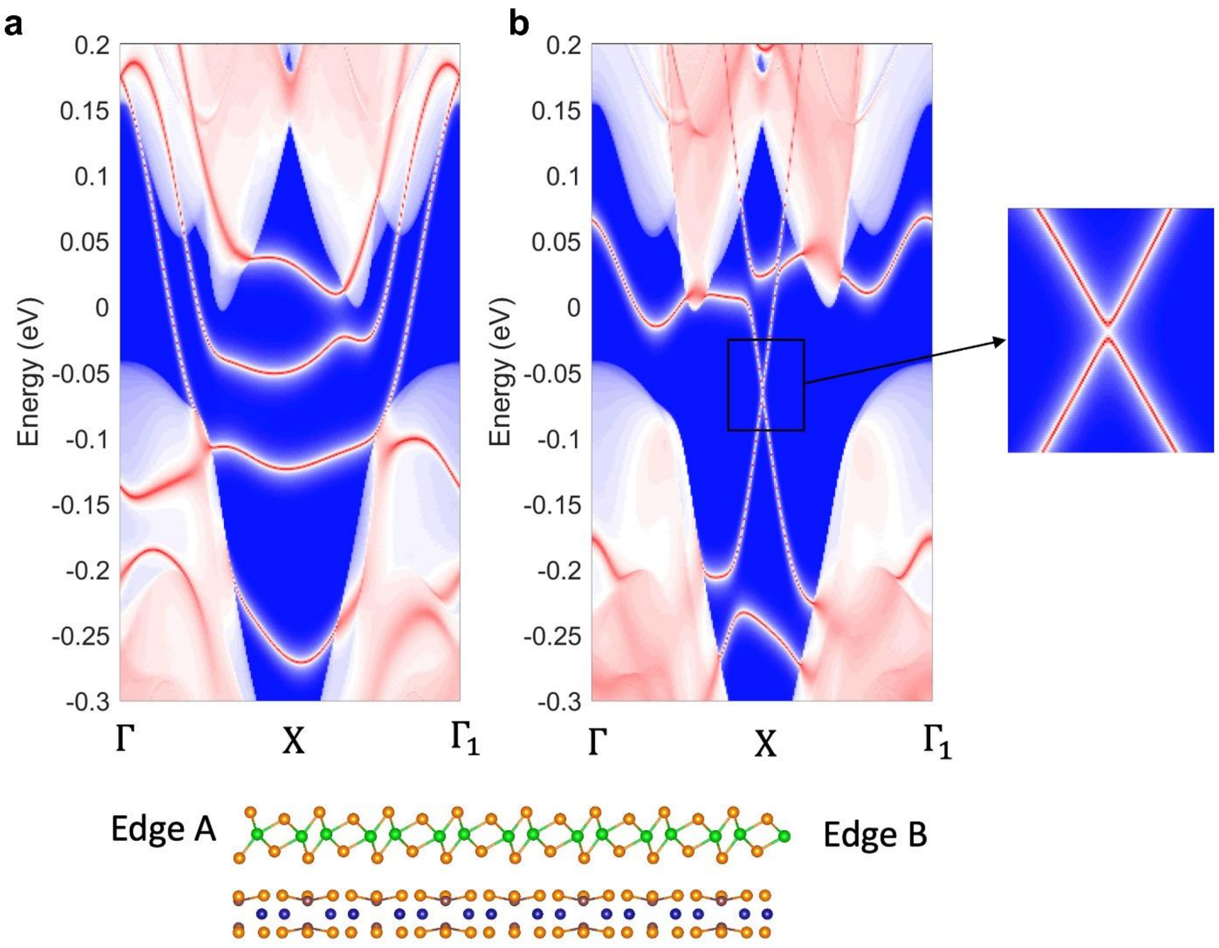

Fig. S8. **Edge band structures for the semi-infinite WTe_2/CGT bilayer**. **a**, **b**, The edge band structures for edge termination A (**a**) and B (**b**) which are shown in the lower panel. The dispersion is from the Γ point to X, and to Γ at the next Brillouin zone (Γ_1). The color from white to red represents the increasing weight of edge states. Blue regions have no state, white and pink regions are bulk states, and red regions represent edge states. Because both time-reversal symmetry and inversion are broken on the edge, the edge dispersions are asymmetric about the X point. The original quantum spin Hall edge states are gapped due to the induced magnetism.

Figure S7a shows the calculated band structure with the Berry curvature (Ω_{xy}) of WTe_2 on CGT substrate. The spin split pair of bands generate different signs of Berry curvature. The lowest conduction bands, compared to the highest valence bands, are more sensitive to the magnetic substrate. The spin splitting of the lowest conduction bands between Γ and X is about

30 meV (see Fig. S7a). Figure S7b shows calculated σ_{xy}^{z} as a function of the chemical potential μ. At low temperatures, the Nernst conductivity α_{xy}^{z} follows the Mott relation:

$$\left.\frac{\alpha_{xy}^{z}}{T}\right|_{T\to 0} = -\frac{\pi^2 k_b^2}{3|e|}\frac{d\sigma_{xy}^{z}}{d\mu} \quad \text{(S4).}$$

Finally, based on the Wannier function Hamiltonian, we compute the edge band structure of a semi-infinite WTe_2/CGT bilayer (Fig. S8). The edge states are gapped out at X and Γ/Γ_1 because of the magnetic proximity. The left-moving and right-moving edge states are asymmetric in the dispersion, because both time-reversal symmetry and inversion symmetry are broken on the edge. There are multiple and complicated edge states because of the existence of dangling bonds on the boundary

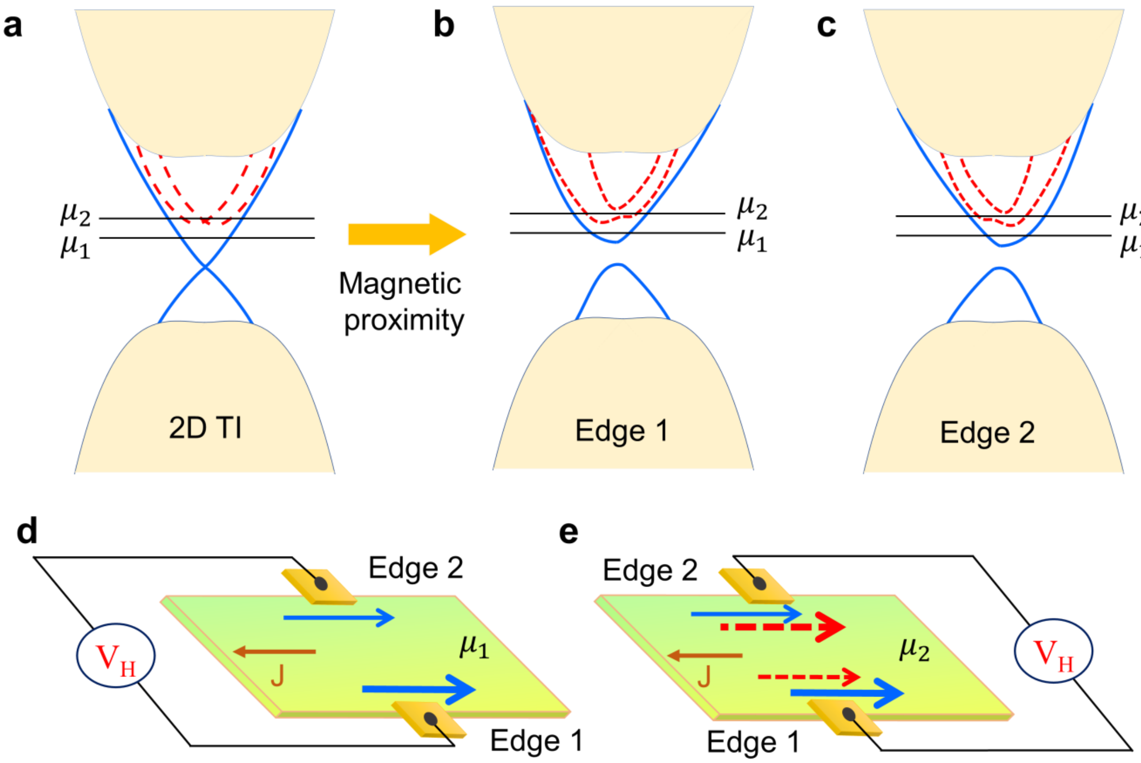

Fig. S9. Schematics of edge states. **a**, Illustration of band structure of two-dimensional topological insulator (2D TI). **b**, **c**, Band structures of the 2D TI at Edge 1, the lower edge (b), and Edge 2, the upper edge (c), of the device shown in (d) and (e) after the magnetic proximity effect is introduced. Blue and red dashed curves represent the Dirac and the ordinary edge states, respectively, and the light-yellow regions are the bulk states. Consequently, the edge dispersions

exhibit asymmetry between Edge 1 (b) and Edge 2 (c). **d**, Edge conductance asymmetry between Edge1 and Edge 2 due to the Fermi velocity asymmetry. For the Fermi level at position μ_1, Edge 1 is more (less) conductive than Edge 2 for the right (left)-flowing current, leading to a different voltage (V_H) between the two edges. The thickness of arrows represents the magnitude of edge current flow. **e**, Edge conductance asymmetry for the Fermi level at position μ_2. Due to the presence of the ordinary edge states (red), there is a competition between conductance at Edge 1 and Edge 2. It is possible to have a sign change for the transverse voltage signal.

9. Gate voltage dependence of 1f resistance and 2f voltage in device D1 at 4 K

Gate voltage (V_g) dependence of resistance contains electronic structure information of materials. We perform 1f and 2f voltage measurements using an AC current with the rms magnitude of 3 μA and the frequency of 13 Hz that is fed into ML-WTe_2 at 4 K. Fig. S10a plots the V_g-dependent 1f resistance from channel 4-7 of device D1 which probes the edge conduction at low temperatures. Only a very broad peak is observed around zero gate voltage, indicating the absence of any measurable gap near the charge neutral point in the edge states, which is consistent with the previous reports (31, 32) and our result presented in the previous section. Fig. S10b summarizes the out-of-plane magnetic field H_z dependence of the 2f voltage signal at selected V_g's. As discussed in the main text, we attribute the 2f voltage signal to ANE because of the Joule heating. It can be clearly seen that the magnitude of ANE hysteresis loop is significantly suppressed under high gate voltages. We summarize the V_g-dependence of magnitude of the 2f voltage hysteresis V_{4-7}^{2f} in Fig. S9c which shows much richer structures than the 1f signal. The peak value at the zero gate voltage, $V_{4-7}^{2f}(0\ V)$, is ~7 times larger than V_{4-7}^{2f} at $\pm 10\ V$. At 4 K, the $V_{4-7}^{2f}(0\ V)$ signal comes from the edge conduction as previously discussed. As V_g is swept to large values on both sides, the Fermi level approaches the bulk bands, and more bulk carriers are involved in the transport. In order to fully understand the detailed V_g-dependence, the Berry curvature of the electronic band need to be calculated, and AHE conductivity, as well as ANE coefficient, need to be studied.

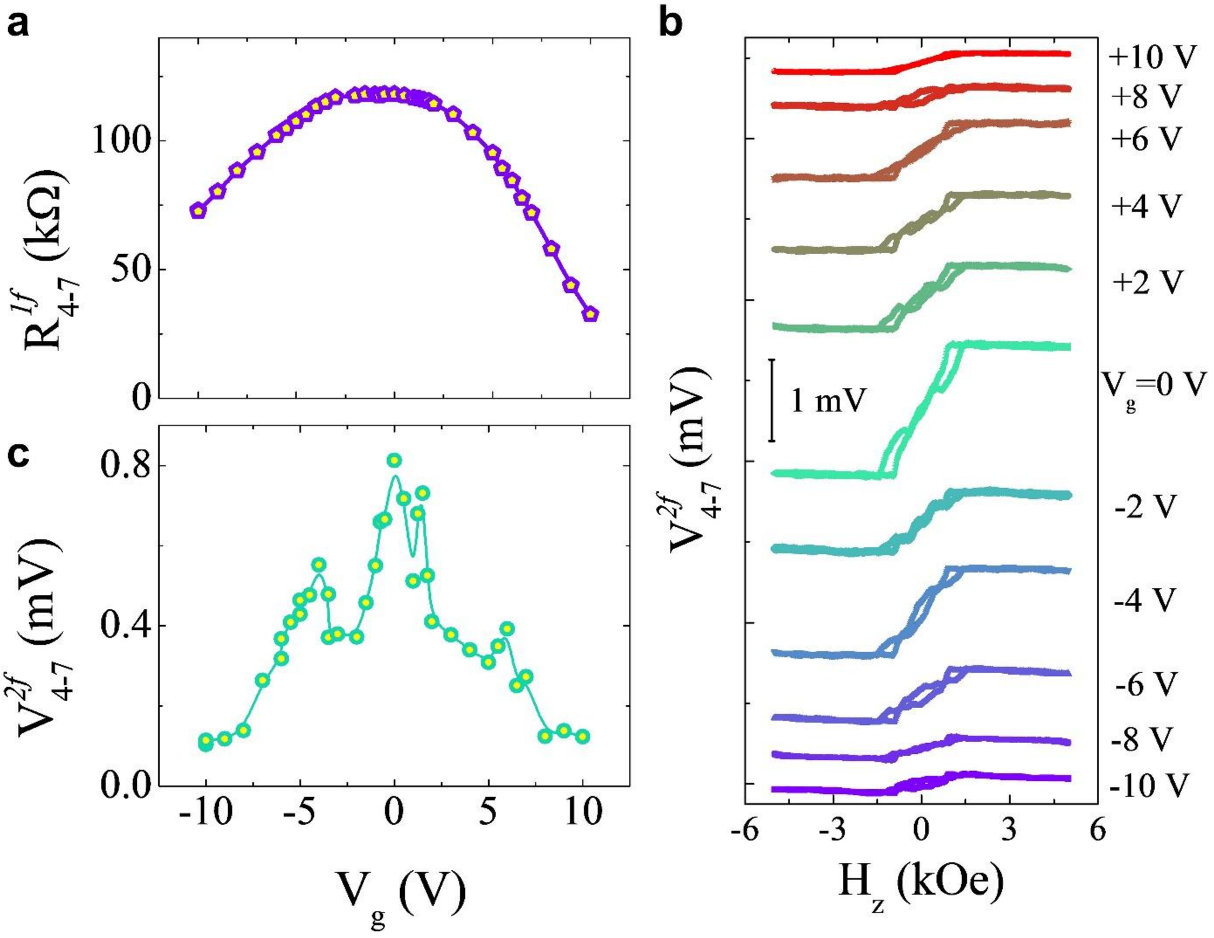

Fig. S10. Gate voltage dependence of 1*f* resistance and 2*f* voltage from 4-7 channel of device D1 at 4 K. **a**, 1f resistance from 4-7 channel of device D1 as a function of bottom gate voltage V_g. **b**, Out-of-plane magnetic field dependence of the 2f voltage at selected gate voltages. V_{4-7}^{2f} is measured while an AC current with rms magnitude of 3 μA and frequency of 13 Hz is fed in 3-8 channel. **c**, Gate voltage dependence of the magnitude of V_{4-7}^{2f}.

10. Separation of bulk and edge ANE signals

The low-temperature ANE signal sign reversal in the Bulk+Edge channel (Fig. 3d) and the absence of the sign reversal in the Bulk-only channel (Fig. 3e) reveal the two-component transport behavior of the ANE in ML-WTe_2/CGT. We can separate the bulk and edge contributions by adopting a simple "parallel battery-resistor" model. As illustrated in Fig. 4a, two

parallel channels, i.e., edge and bulk, are considered, each consisting of a battery with voltage V^{ANE} and a resistor with conductance G. The total voltage V_{B+E}^{ANE} from the mixed channel reads as

$$V_{B+E}^{ANE} = \frac{I_{B+E}}{G_{B+E}} = \frac{V_E^{ANE} G_E + V_B^{ANE} G_B}{G_E + G_B} \quad (S5),$$

where V_E^{ANE} (V_B^{ANE}) refers to the ANE voltage from the edge (bulk) channel, G_E(G_B) is the conductance of the edge (bulk) channel. I_{B+E} is the total current in the mixed channel. The total conductance from both edge and bulk channels reads as

$$G_{B+E} = G_B + G_E \quad (S6)$$

One can readily see in Eq. S5 that $V_{B+E}^{ANE} \approx V_E^{ANE}$ when the edge conduction dominates at low temperatures ($G_E \gg G_B$). We measure G_{B+E}, G_B, V_{B+E}^{ANE} and V_B^{ANE} in our experiments; therefore, we can calculate G_E, V_E^{ANE} from Eqs. S5 and S6. After disentangling the contributions from the edge and bulk channels, we plot them in Fig. 4b.

11. Effect of low-temperature thermal conductivity on ANE magnitude

The P-normalized ANE signals from channels 5-6 and 13-14 in device D7 become significantly larger at low temperatures, a trend contradictory to the third law of thermodynamics. We attribute this apparent increase in both channels to a rapidly decreasing thermal conductivity κ at low temperatures, which greatly enlarges the actual ∇T since $\nabla T \propto \frac{P}{\kappa}$. Larger ∇T in turn leads to an enhanced ANE voltage.

The thermoelectric coefficients S are defined by $E = S\nabla T$, where $\boldsymbol{E}$ is the electrical field produced by the temperature gradient and S the thermoelectric tensor. To understand how thermoelectric transport behaves, it is appropriate to taking thermal conductivity into the account. Thus, we consider the $\frac{\Delta V_{ANE}}{|\nabla T|} \propto \frac{\Delta V_{ANE}\kappa}{P}$. If we crudely assume that the thermal conductivity is dominated by phonons in the surrounding materials, i.e., $\kappa \sim T_s^3$ at low temperatures. For constant heating power, we should multiply the ANE voltage signal by phonon thermal conductivity, i.e., $\frac{\Delta V_{ANE}}{\nabla T} \propto \frac{\Delta V_{ANE} T_S^3}{P}$. As shown in Fig. S11, we find that both curves approach

zero as T_s → 0 after they are multiplied by T_s^3, which is consistent with the expectation from the thermodynamic third law. Above all, we have explained the diverging trend of the $\frac{\Delta V_{ANE}}{P}$ at low temperatures by considering the effect of the vanishing low-temperature thermal conductivity.

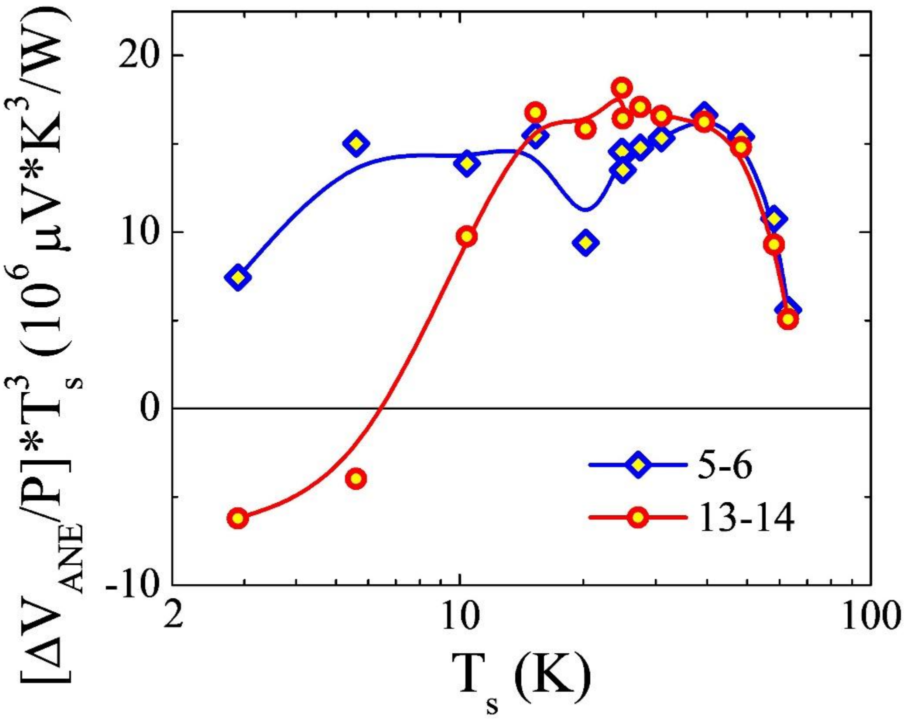

Fig. S11. Replot of the low-temperature ANE signals. The T_s^3 temperature dependence of the phonon thermal conductivity is considered for low temperatures by multiplying the anomalous Nernst effect (ANE) signals from Bulk+ Edge channel (13-14) and Bulk-only channel (5-6) by T_s^3. T_s is the sample temperature.

References

1. Jiang, Y.et al. Raman Fingerprint of Semi-Metal WTe_2 from Bulk to Monolayer. Sci. Rep. **6**, 19624 (2016).
2. Kehlberger, A. et al. Length scale of the spin Seebeck effect. Phys. Rev. Lett. **115**, 096602 (2015).
3. Saitoh, E., Ueda, M., Miyajima, H. & Tatara, G. Conversion of spin current into charge current at room temperature: Inverse spin-Hall effect. Appl. Phys. Lett. **88**, 182509 (2006).
4. Sinova, J., Valenzuela, S. O., Wunderlich, J., Back, C. H. & Jungwirth, T. Spin Hall effects. Rev. Mod. Phys. **87**, 1213–1260 (2015).
5. Mason, S. et al. Violation of the Wiedemann−Franz law through reduction of thermal conductivity in gold thin films. Phys. Rev. Mater. **4**, 065003 (2020).
6. Lin, G. T. et al. Tricritical behavior of the two-dimensional intrinsically ferromagnetic semiconductor $CrGeTe_3$. Phys. Rev. B **95**, 245212 (2017).
7. Fugallo, G. et al. Thermal conductivity of graphene and graphite: collective excitations and mean free paths. Nano Lett. **14**, 6109–6114 (2014).
8. Pop, E., Varshney, V. & Roy, A. K. Thermal properties of graphene: fundamentals and applications. MRS Bull. **37**, 1273–1281 (2012).
9. Xiao, Y. et al. Specific heat and quantized thermal conductance of single-walled boron nitride nanotubes. Phys. Rev. B **69**, 205415 (2004).
10. Glazov, V. M. and Pashinkin, A. S. The thermophysical properties (heat capacity and thermal expansion) of single-crystal silicon. High Temp. **39**, 413 (2001).
11. Asheghi, M. et al. Temperature-dependent thermal conductivity of single-crystal silicon layers in SOI substrates. J. Heat Transfer **120**, 30–36 (1998).
12. Sichel, E. K. et al. Heat capacity and thermal conductivity of hexagonal pyrolytic boron nitride. Phys. Rev. B **13**, 4607 (1976).
13. Glassbrenner, C. J. and Slack, G. A. Thermal conductivity of silicon and germanium from 3 K to the melting point. Phys. Rev. **134**, A1058 (1964).
14. Westrum, E. Determination of the low-temperature heat capacity of vitreous silicon dioxide, or quartz and of cristobalite. In: Final report, project 2148, Owens Illinois Glass Company, Toledo, Ohio, Engineering Research Inst, Univ Michigan, Ann Harbor (1954).

15. Geballe, T. H. and Giauque, W. F. The heat capacity and entropy of gold from 15 to 300°K. J. Am. Chem. Soc. **74**, 2368 (1952).

16. Brown, D. B. et al. Thermal boundary conductance and phonon transmission in hexagonal boron nitride/graphene heterostructures. Phys. Status Solidi A **216**, 1900446 (2019).

17. Liu, Y. et al. Thermal conductance of the 2D MoS_2/h-BN and graphene/h-BN Interfaces. Sci Rep **7**, 43886 (2017).

18. Li, X. et al. Thermal conduction across a boron nitride and SiO_2 interface. J. Phys. D: Appl. Phys. **50**, 104002 (2017).

19. Ong, Z.-Y. et al. Controlling the thermal conductance of graphene/h−BN lateral interface with strain and structure engineering. Phys. Rev. B **93**, 075406 (2016).

20. Kato, R. et al. Development of a Frequency-Domain Method Using Completely Optical Techniques for Measuring the Interfacial Thermal Resistance between the Metal Film and the Substrate. Jpn. J Appl. Phys. **50** (10R), 106602 (2011).

21. Käding, O. W. et al. Thermal conduction in metallized silicon-dioxide layers on silicon. Appl. Phys. Lett. **65**, 1629 (1994).

22. Nakayama, H. et al. Spin Hall magnetoresistance induced by a nonequilibrium proximity effect. Phys. Rev. Lett. 110, 206601 (2013).

23. Lohmann, M. et al. Probing magnetism in insulating Cr2Ge2Te6 by induced anomalous Hall effect in Pt. Nano Lett. 19, 2397–2403 (2019).

24. Wang, Z., Tang, C., Sachs, R., Barlas, Y. & Shi, J. Proximity-induced ferromagnetism in graphene revealed by the anomalous Hall effect. Phys. Rev. Lett. 114, 016603 (2015).

25. Tang, C. et al. Above 400-K robust perpendicular ferromagnetic phase in a topological insulator. Sci. Adv. 3, e1700307 (2017).

26. Yao, X. et al. Record High-Proximity-Induced Anomalous Hall Effect in (BixSb1−x)2Te3 Thin Film Grown on CrGeTe3 Substrate. Nano Lett. 19, 4567 (2019).

27. Zhao, W. et al. Magnetic proximity and nonreciprocal current switching in a monolayer WTe_2 helical edge. Nat. Mater. **19**, 503–507 (2020).

28. Perdew, J. P., Burke, K. & Ernzerhof, M. Generalized Gradient Approximation made simple. Phys. Rev. Lett. **77**, 3865–3868 (1996).

29. Kresse, G. & Joubert, D. From ultrasoft pseudopotentials to the projector augmented-wave method. Phys. Rev. B **59**, 1758–1775 (1999).

30. Grimme, S. Semiempirical GGA-type density functional constructed with a long-range dispersion correction. J. Comput. Chem. **27**, 1787–1799 (2006).

31. Fei, Z. et al. Edge conduction in monolayer WTe_2. Nat. Phys. **13**, 677–682 (2017).

32. Wu, S. et al. Observation of the quantum spin Hall effect up to 100 kelvin in a monolayer crystal. Science **359**, 76–79 (2018).
